# The role of NLRP3 inflammasome in aging and age-related diseases

**DOI:** 10.1186/s12979-023-00395-z

**Published:** 2024-02-05

**Authors:** Ruikai Liang, Xinrui Qi, Qi Cai, Liyan Niu, Xi Huang, Deju Zhang, Jitao Ling, Yuting Wu, Yixuan Chen, Pingping Yang, Jianping Liu, Jing Zhang, Peng Yu

**Affiliations:** 1 Department of Endocrinology and Metabolism, The Second Affiliated Hospital of Nanchang University, Jiangxi, Nanchang, China; 2 The Second Clinical Medical College of Nanchang University, The Second Affiliated Hospital of Nanchang University, Jiangxi, Nanchang, China; 3 Queen Mary School, Nanchang University, Nanchang, China; 4 Huan Kui College of Nanchang University, Nanchang, China; 5 Food and Nutritional Sciences, School of Biological Sciences, The University of Hong Kong, Hong Kong, China; 6 Department of Anesthesiology, The Second Affiliated Hospital of Nanchang University, Nanchang, Jiangxi Province China

**Keywords:** NLRP3 inflammasome, Aging, Related diseases, Regulatory mechanisms, Therapeutic strategies

## Abstract

The gradual aging of the global population has led to a surge in age-related diseases, which seriously threaten human health. Researchers are dedicated to understanding and coping with the complexities of aging, constantly uncovering the substances and mechanism related to aging like chronic low-grade inflammation. The NOD-like receptor protein 3 (NLRP3), a key regulator of the innate immune response, recognizes molecular patterns associated with pathogens and injury, initiating an intrinsic inflammatory immune response. Dysfunctional NLRP3 is linked to the onset of related diseases, particularly in the context of aging. Therefore, a profound comprehension of the regulatory mechanisms of the NLRP3 inflammasome in aging-related diseases holds the potential to enhance treatment strategies for these conditions. In this article, we review the significance of the NLRP3 inflammasome in the initiation and progression of diverse aging-related diseases. Furthermore, we explore preventive and therapeutic strategies for aging and related diseases by manipulating the NLRP3 inflammasome, along with its upstream and downstream mechanisms.

## Introduction

The aging of the global population is increasingly apparent, with current epidemiological studies indicating that 11% of the world's population is aged over 60, projected to rise to 22% by 2050 [1]. This demographic shift presents unprecedented challenges to public health [2]. Aging is a multifaceted process involving multiple organs and tissues, leading to the functional degeneration of various cell types [3]. It is considered a major risk factor for chronic diseases like cardiovascular disease, diabetes and even fatal cancer, which seriously affects the health status of the elderly [1].

The accumulation of senescent cells is closely related to aging. Senescent cells are a class of cells that undergo irreversible cell cycle arrest following cellular stress or injury. Despite ceasing division, these cells remain metabolically active and exhibit pro-inflammatory characteristics [4]. Systemic inflammation associated with senescent cells is thought to exacerbate vascular disease, contributing to atherosclerosis, increased cortisol secretion, and bone resorption [1]. Aging of the immune system is also a major feature of the aging process, marked by diminished immune function, declining naïve T cells, heightened memory T cells, cytokine production shifts, and increased low-grade inflammation [5], rendering older adults more susceptible to diseases and resulting in tissue failure and death [2, 3].

The inflammasome, a multiprotein complex central to inflammation-related diseases, is particularly exemplified by the NOD-like receptor protein 3 (NLRP3) inflammasome. Activated by various stimuli, the NLRP3 inflammasome takes part in the regulation of inflammatory responses and has been implicated in various age-related chronic diseases. Inhibiting its activation holds promise for preventing disease and preserving tissue function [6–9]. This paper explores the mechanistic interplay of NLRP3 and its association with aging-related diseases, aiming to establish a theoretical foundation for utilizing NLRP3 in the treatment of age-related conditions.

## Aging and age-related diseases

### Overview of aging

**Fig. 1:**
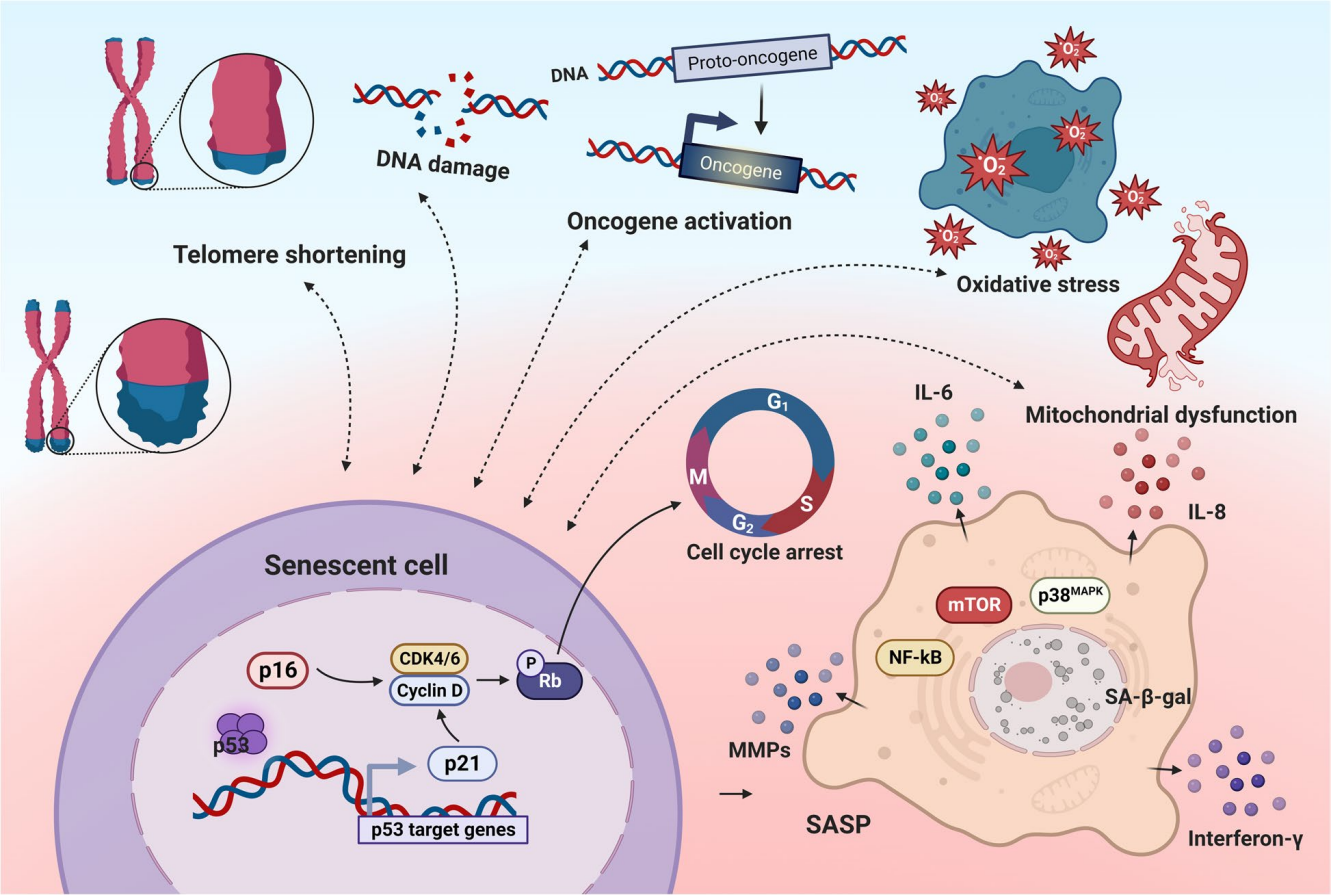
Mechanisms and characteristics of cellular senescence. CDK, cyclin-dependent kinase; IL-6, interleukin-6; IL-8, interleukin-8; MMPs, matrix metalloproteinases; mTOR, mechanistic target of rapamycin; Rb, Retinoblastoma protein; SASP, senescence-associated secretory phenotype; SA-β-gal, senescence-associated β-galactosidase

Cellular senescence is a comprehensive manifestation of the decline and disruption of physiological functions during the degenerative period, induced by various factors such as telomere dysfunction, DNA damage, oncogene activation, oxidative stress, and mitochondrial dysfunction [10] (Fig. 1). One of the key features of cellular senescence is the permanent withdrawal of cells from the cell cycle. The p53/p21 and p16/Rb tumor suppressor gene pathways regulate cell cycle arrest (Fig. 1). Specifically, during continuous cell proliferation, telomere attrition triggers a permanent DNA damage response, activating the tumor suppressor gene p53 [11]. Subsequently, p53 upregulates the expression of the transcriptional target p21. The activated p21 inhibits cyclin-dependent kinase (CDK)-mediated Rb phosphorylation, preventing the cell cycle from progressing [12]. Additionally, p16 is induced in senescent cells, blocking CDK4/CDK6-mediated Rb phosphorylation and contributing to the cellular senescence phenomenon [13]. The programmed death going with senescent cells could serve as an effective anti-cancer mechanism by blocking tumor cells from the cell cycle. Although senescent cells stop proliferating, they maintain metabolic activity for a certain period of time, just not as efficiently as when they were younger. For example, cells undergoing senescence show significant changes in their secretome, leading to chronic low-grade inflammation and disease in the organism, which is termed the senescence-associated secretory phenotype (SASP) [12]. Main elements of SASP are the pro-inflammatory cytokines interleukin-6 (IL-6), IL-8, interferon-γ (IFN-γ) and matrix metalloproteinases [14]. Most SASP components are regulated by NF-kB, p38^MAPK^ and mechanistic target of rapamycin (mTOR) signaling [15] (Fig. 1).

Thus, cell cycle-related factors such as p16, p21, p53, Rb and related factors secreted by senescent cells can be used as biomarkers of cellular senescence [11, 12]. Additionally, markers of cellular senescence still include the massive accumulation of Senescence-Associated β-Galactosidase (SA-β-gal) due to heightened lysosomal content and activity, downregulation of nuclear fibrillar protein B1 (lamin B1) caused by alterations in the nuclear envelope, and increased lipofuscin, detectable by Sudan Black B staining [12]. Morphologically, senescent cells exhibit enlarged cytostomes with irregular shapes and altered plasma membrane composition [16]. 

Notably, pro-inflammatory markers in aging organisms register levels that are 2–4 times higher than those in younger individuals [17], and inflammation can lead to cellular senescence by causing oxidative damage, damaging DNA, and many other aspects such as mitochondrial dysfunction and dysregulation of the ubiquitin–proteasome system [18]. These factors are interrelated and affect each other: oxidative damage promotes cells to secrete more pro-inflammatory factors (tumor necrosis factor-α (TNF-α), IL-1β, IL-2, and IL-6), establishing a chronic inflammatory microenvironment; the inflammatory microenvironment in turn exacerbates oxidative damage, inducing the production of more reactive oxygen species (ROS) (hydrogen peroxide and free radicals), which decrease the ability to repair damage and accelerate the rate of chromosomal telomere shortening [19].

### Age-related diseases

While aging is a physiological process, it is often accompanied by various co-morbidities. Cellular senescence, is linked to an excessive inflammatory response, contributing to numerous age-related diseases such as cardiovascular disease, osteoporosis, cataracts, diabetes, and Alzheimer's. Inflammation also plays a decisive role in the onset, development, invasion, and metastasis of cancer. This emphasizes that our focus should not solely be on directly intervening in aging but rather on preventing or alleviating age-related diseases. Effective control of inflammation becomes a key strategy in addressing both aging-related complications and diseases.

In atherosclerosis, the expression of antioxidant enzymes (superoxide dismutase and catalase) is down-regulated in aging endothelial cells, and the DNA repair capacity is reduced, leading to a long-term chronic oxidative stress state in vascular endothelial cells and rapid apoptosis respectively [20, 21]. The same mechanism also occurs in the lens epithelial cells of cataract patients, which eventually cause the loss of lens epithelial cell viability and death [22]. Moreover, increased expression of p16 was identified in pancreatic β-cells from patients with type 2 diabetes mellitus (T2DM), in osteoblasts from individuals with osteoporosis, and in astrocytes, cells responsible for modulating synaptic function and clearing metabolic byproducts [23], in Alzheimer's patients. These findings suggest a potential association with blunted insulin synthesis in T2DM, accelerated aging of osteoblasts in osteoporosis, and neuronal damaging in Alzheimer's disease, respectively [24, 25]. Farr et al. also found that SASP factors such as IL-6, IL-8, membrane cofactor protein-1 in the bone microenvironment cause diminished differentiation capacity of bone progenitor cells and increased osteoclast production, leading to an imbalance in bone homeostasis [26]. Immune imbalance and elevated levels of oxidative stress are also observed [27]. Consistently, brain aging is characterized by decreased glucose use, mitochondrial dysfunction, and excessive oxidative stress. In vitro assays have shown that astrocytes undergo senescence in response to ROS exposure and increased SASP factors are produced by activated astrocytes [25]. Microglia from aged mice showed telomere shortening and DNA damage [25]. The intricate interplay of inflammatory factors and ROS levels contributes to age-related disease and underscores their significance in disease progression.

## The role and regulatory mechanisms of NLRP3 inflammasomes in aging

Immunosenescence, characterized by the declining ability of the immune system to effectively respond to pathogens and cancer cells during aging, is a critical aspect of age-related changes. Chronic low-grade inflammation has been consistently associated with immunosenescence. Senescent cells exhibit a SASP, releasing various pro-inflammatory factors and chemokines, thereby initiating an inflammatory response that affects the surrounding tissues [28]. In this context, we explore the role of NLRP3 inflammasomes and their regulatory mechanisms in the aging process.

**Fig. 2:**
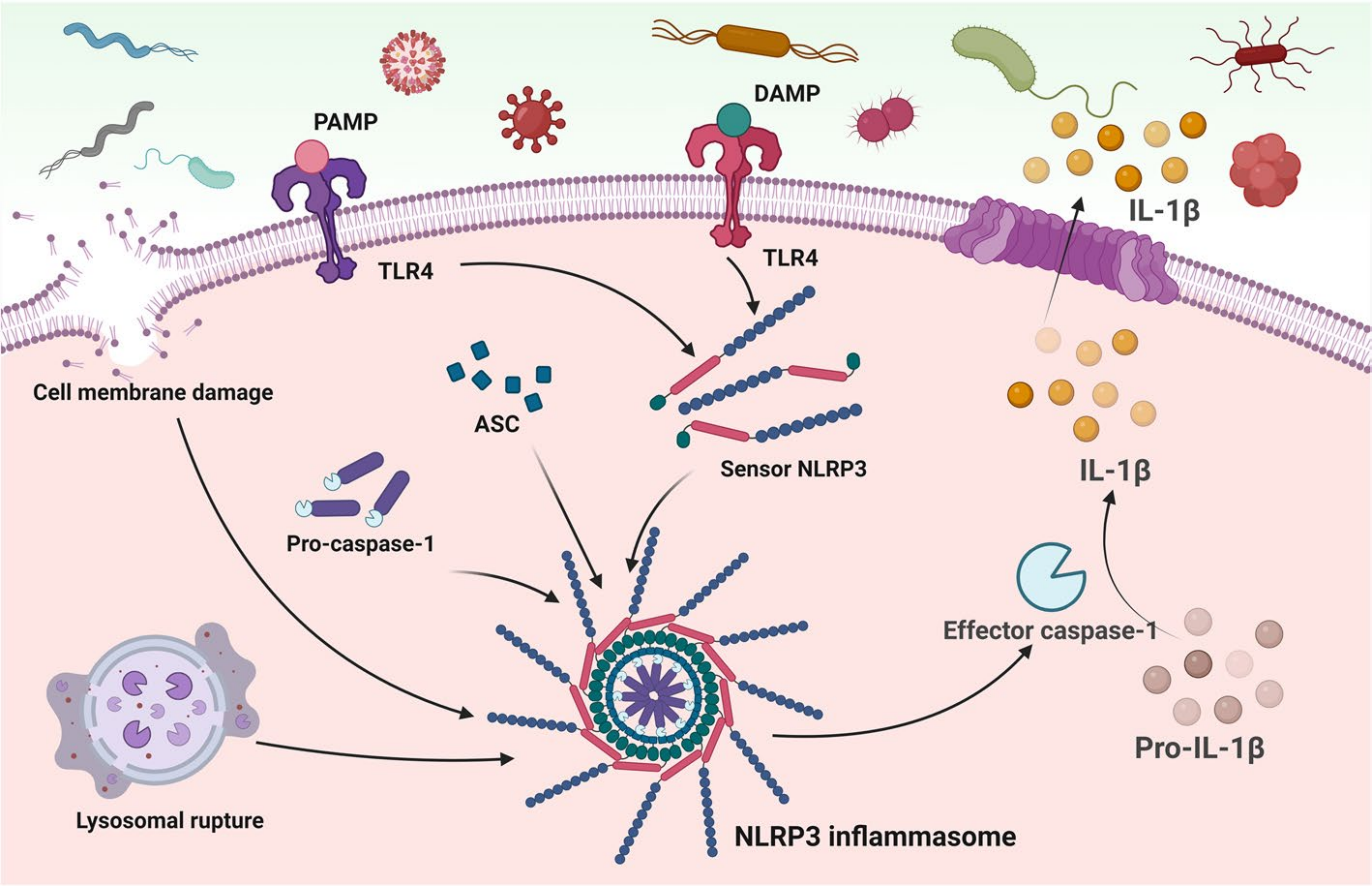
Two-step activation of NLRP3 inflammasome at the molecular level. ASC, apoptosis-associated speck-like protein; DAMP, damage-associated molecular pattern; IL-1β, interleukin-1β; NLPR3, NOD-like receptor protein 3; PAMP, pathogen-associated molecular pattern; pro-IL-1β, pro-interleukin-1β; TLR4, Toll-like receptor 4

The intricate process of inflammation is intricately linked to the activation of NLRP3 inflammasomes, comprising sensors (NLRP3), adaptors (apoptosis-associated speck-like protein (ASC)), and effectors (caspase-1) [29]. This multiprotein complex is activated through a two-step mechanism [30]. Initially, cells recognize signals from pathogen-associated molecular patterns (PAMPs) or damage-associated molecular patterns (DAMPs) via receptors like Toll-like receptor 4 (TLR4), initiating the synthesis of sensors, primarily NLRP3. In the subsequent step, various cellular events, such as membrane damage or lysosomal rupture, facilitate the formation of the NLRP3 inflammasome complex. Throughout this process, NLRP3 undergoes de-ubiquitination and associates with ASC, which binds to pro-caspase-1, resulting in the formation of a large multimeric protein complex—the NLRP3 inflammasome. Active caspase-1, an effector produced by this complex, cleaves pro-IL-1β into its mature and secreted form, IL-1β, thereby initiating a potent inflammatory response (Fig. 2).

Numerous studies have explored the potential involvement of NLRP3 inflammasome in the aging process. For instance, Christina D. Camell et al. demonstrated an increase in adipose-associated lymphoid cluster formation and expansion of non-senescent adipose B cells and splenic age-associated B cells with age [31]. The inhibition of NLRP3 inflammasome accumulation was observed to mitigate these age-related phenomena, suggesting a role for NLRP3 inflammasome in promoting metabolic damage during aging [31]. In a 2021 animal study, NLRP3 inflammasome expression was found to be elevated in the ovaries of reproductively senescent mice. Conversely, the use of the NLRP3 inflammasome inhibitor MCC950 increased ovarian pregnancy rates, indicating a potential mediation of the aging process in the female reproductive system by NLRP3 inflammasome [32]. Notably, certain studies, such as those by Li et al., have indicated that activation of the NLRP3 inflammasome in mice could enhance local inflammatory responses in blood vessels, thereby accelerating the development of atherosclerosis—a critical manifestation of vascular aging [33]. Not only in the cardiovascular system, the NLRP3 inflammasome has also been implicated in promoting the occurrence and progression of bone and joint diseases, which are recognized manifestations of individual aging [34]. The provided examples illuminate different aspects of the potential involvement of NLRP3 inflammasome in aging; however, it is crucial to emphasize that more experimental data are required for conclusive insights into the role of NLRP3 inflammasome in the aging process.

## The role and regulatory mechanisms of NLRP3 inflammasomes in aging-related disease

### Cardiovascular disease

Much epidemiological evidence suggests that inflammation mediated by inflammatory molecules, including the NLRP3 inflammasome, is a powerful risk factor for cardiovascular disease.

**Fig. 3:**
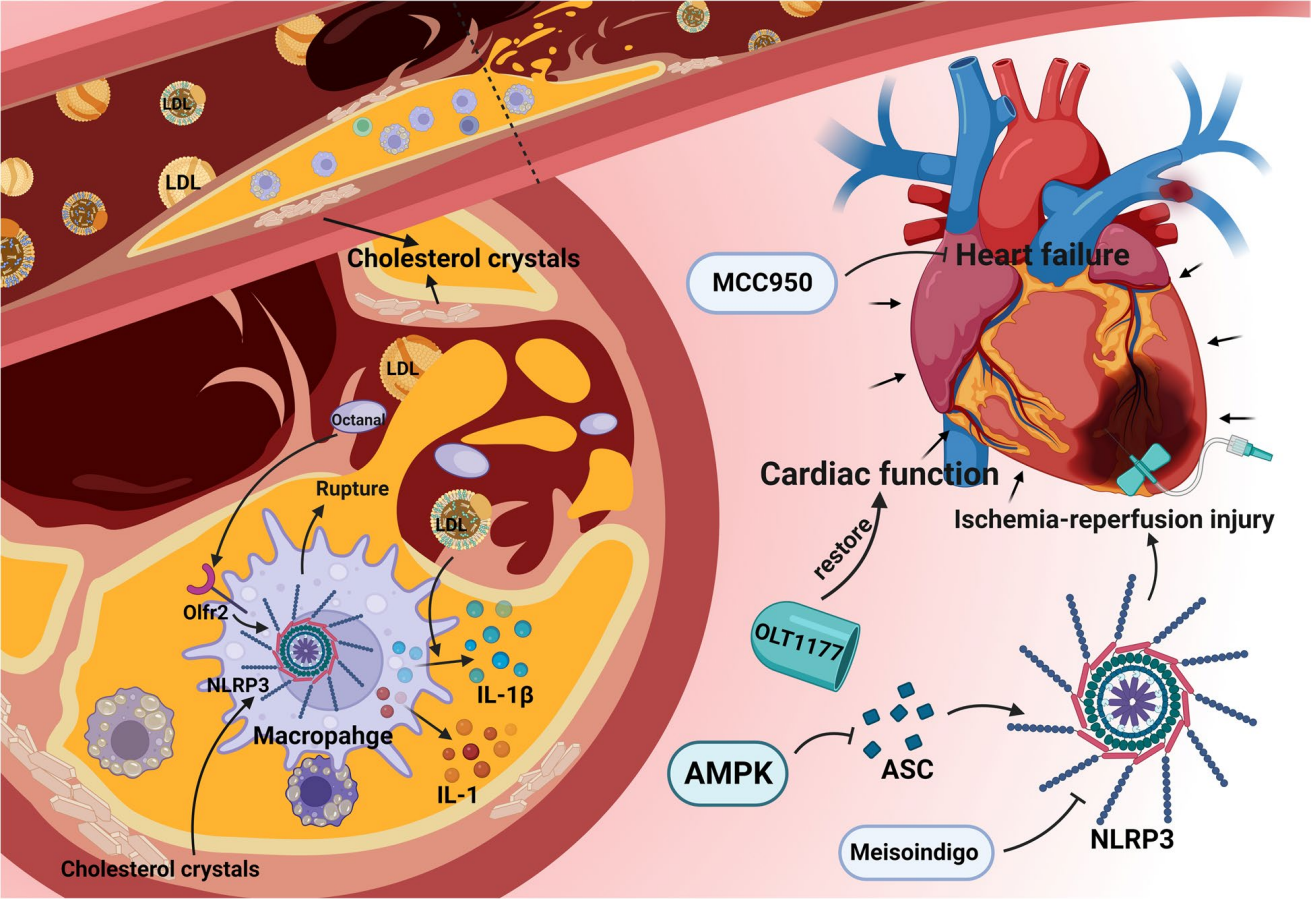
Activation of NLRP3 inflammasome in cardiovascular disease. AMPK, AMP-activated protein kinase; ASC, apoptosis-associated speck-like protein; IL-1, interleukin-1; IL-1β, interleukin-1β; LDL, low density lipoprotein; Olfr2, olfactory receptor 2

Atherosclerosis is an important manifestation of cardiovascular aging, as cells having markers of aging can be found in advanced atherosclerotic plaques. There is increasing evidence that inflammation is a crucial factor leading to atherosclerosis. The process of atherosclerosis is influenced by endogenous molecules, such as cholesterol crystals present in damaged vascular endothelial cells and other substances derived from damaged or necrotic tissue. These endogenous danger signals activate the NLRP3 inflammasome in macrophages, ultimately leading to the rupture of fragile atherosclerotic plaques, leading to thrombosis (Fig. 3) [35]. In addition, animal experiments in mice have found that the role of the NLRP3 inflammasome in atherosclerosis is due to its effector cytokine IL-1β. After feeding low density lipoprotein receptor knockout model mice with a high-fat diet for a period, it was found that their levels of IL-1β and the incidence of atherosclerosis were significantly higher than those of normal mice (Fig. 3). Loss of the low-density lipoprotein receptor results in the inability of cells to take up cholesterol in the plasma, thereby increasing plasma cholesterol concentrations. This suggests that the NLRP3 inflammasome can be activated by cholesterol in plasma [29]. A study by Marco Orecchioni et al. found that mouse plasma containing octanal activates the olfactory receptor Olfr2 expressed by vascular macrophages, driving atherogenesis through NLRP3-dependent IL-1 production (Fig. 3) [36].

Cardiac ischemia–reperfusion injury is also associated with the occurrence of inflammatory responses (Fig. 3) [37]. In an experiment with metformin, the researchers found that AMPK, a protein kinase, can reduce myocardial ischemia–reperfusion injury by inhibiting the expression of the NLRP3 inflammasome and ASC [38]. Likewise, Meisoindigo is demonstrated to suppress NLRP3 inflammasome production, exhibiting a pivotal role in diminishing ischemia–reperfusion injury [39]. Collectively, these findings support the involvement of the NLRP3 inflammasome in promoting ischemia–reperfusion injury.

As an additional manifestation of aging, chronic heart failure in humans is intricately linked to inflammation, a relationship substantiated through animal model experiments. In a mouse model with Tet2 mutation [40], the NLRP3 inflammasome inhibitor MCC950 was administered, revealing that MCC950 prevented heart failure development (Fig. 3). Moreover, it eliminated disparities in cardiac parameters between Tet2-deficient and wild-type mice, ultimately reversing cardiac fibrosis and hypertrophy [40]. In another set of mouse experiments, OLT1177, an inhibitor of the NLRP3 inflammasome, partially restored cardiac function that was compromised in heart failure (Fig. 3) [41]. Despite these promising findings associating heart failure with inflammation, the majority of anti-inflammatory drugs used in clinical trials did not demonstrate efficacy in preventing heart failure [42–45]. This lack of success may stem from the intricate immune cell cascade that generates multiple pro-inflammatory mediators, resulting in the limited effectiveness of anti-inflammatory treatments [46]. Several other cardiovascular diseases, such as hereditary structural cardiomyopathy and idiopathic dilated cardiomyopathy, are also associated with NLRP3 inflammasome activation [47]. The involvement of NLRP3 inflammasome in the advancement of myocardial dysfunction and non-ischemic dilated cardiomyopathy has been observed, primarily through cardiomyocyte apoptosis mediated by a cysteine asparaginase-1-dependent mechanism [48].

In conclusion, cardiovascular system diseases are associated with the activation of NLRP3 inflammasome. However, there is a lack of established clinical drugs specifically targeting NLRP3 inflammasome to manage cardiovascular diseases. Further investigations into the relationship between NLRP3 inflammasome inhibitory drugs and the progression of cardiovascular diseases are warranted to advance the development of pertinent clinical therapeutics.

### Cancer

**Fig. 4:**
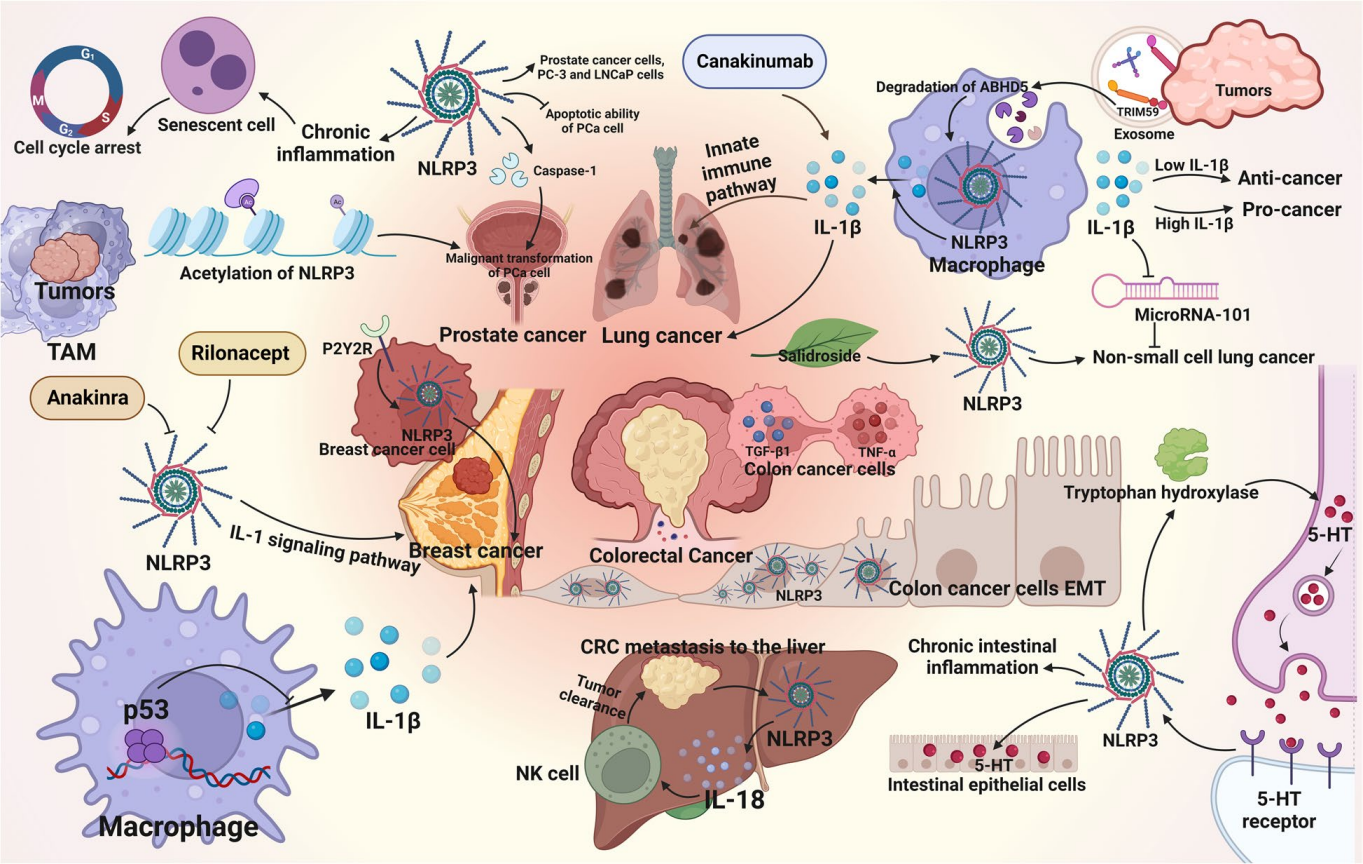
Association of NLRP3 inflammasome with cancer progression. 5-HT, 5-hydroxytryptamine; ABHD5, α/β-Hydrolase domain-containing protein 5; CRC, colorectal cancer; EMT, epithelial-mesenchymal transition; IL-18, interleukin-18; IL-1β, interleukin-1β; LNCaP, lymph node carcinoma of the prostate; P2Y2R, P2Y purinergic receptor 2; PCa, prostate cell; PC-3, prostate cancer cell line; TAM, tumor-associated macrophage; TGF-β1, transforming growth factor-β1; TNF-α, tumor necrosis factor-α; TRIM59, tripartite motif-containing 59

The relationship between cell senescence and cell carcinogenesis is bidirectional. On one hand, cellular senescence induces cell cycle arrest, which can reduce damage to cells during mitosis, such as cancerous cells caused by genetic mutations. Therefore, cellular senescence is an important barrier to prevent cell carcinogenesis to a certain extent [14]. On the other hand, some inflammatory factors like the NLRP3 inflammasome and some other chemokines like IL-6 and IL-8, produced by senescent cells create a conducive growth environment for neighboring cells in a precancerous state that can progress into tumor cells, accelerating their carcinogenesis process [49]. Under the dual effect of cell senescence, the fate of cells depends on the characteristics of the cells themselves and the different external stimuli that impact how cells respond to the senescent process. The developments of different cancers are presented in Fig. 4.

#### Prostate cancer

Xu et al. demonstrated that NLRP3 inflammasome significantly enhanced the proliferation and migration of human prostate cancer cells, prostate cancer cell (PC) line 3 and lymph node carcinoma of the prostate (LNCaP) cells, but decreased the apoptotic ability of PCa cell lines by cell counting kit-8 (CCK-8), TdT-mediated dUTP nick end labeling (TUNEL) and Transwell assays [50]. Their further studies revealed that NLRP3 inflammatory vesicles promote malignant transformation of PCa through activation of caspase-1, providing new possible prognostic biomarkers and potential therapeutic targets for PCa [50]. The study by Zhao et al. also showed that chronic inflammation triggered by the NLRP3 inflammasome is one of the crucial factors leading to the occurrence and development of cancer. They also achieved the effect of directly using NLRP3 inhibitors on cells by interfering with the acetylation of NLRP3, that is, inhibiting the malignant progression of PCa [51].

However, while chronic inflammation caused by NLRP3 inflammasome promotes the occurrence and development of cancer, there is also evidence that these inflammatory factors can induce cell cycle arrest by promoting cell senescence to inhibition [52]. Studies supporting this view have shown that tumor development is inseparable from the microenvironment provided by tumor-associated macrophages (TAM). Re-educated TAM to adopt a pro-inflammatory and anti-tumor phenotype shifts the originally pro-cancer inflammatory factors into anti-cancer agents, thereby suppressing cancer development [52, 53].

The above findings underscore the role of chronic inflammation in PCa induction. Integrated with NLRP3 inflammasome research, targeted pharmacological interventions can alter cell phenotypes promoting cancer, inducing a positive shift towards cell senescence. Simultaneously, this intervention inhibits cancer onset and development, offering a novel treatment idea for PCa. The regulatory mechanism linked with NLRP3 may become a focal point for future research.

#### Lung cancer

In the study by Liang et al., it was observed that tripartite motif-containing 59 (TRIM59) released by tumors is expressed in exosomes. Through the regulation of the proteasomal degradation of Alpha–beta hydrolase domain-containing 5 (ABHD5), macrophages acquire tumor-promoting functions. These altered macrophages activate the NLRP3 inflammasome signaling pathway, leading to an upregulation in the secretion of IL-1β. This process promotes the formation of an inflammatory microenvironment and facilitates cancer metastasis to the lungs, ultimately contributing to the development of lung cancer [54]. In another experiment, researchers discovered the pivotal role of IL-1β in the development of inflammation-induced lung tumors. The mechanism involves IL-1β facilitating the proliferation and migration of non-small cell lung cancer cells by mediating the inhibition of microRNA-101, which has anti-tumor effects [55]. Additionally, data from a clinical trial suggest that canakinumab, a therapeutic agent targeting the IL-1β innate immune pathway, holds significant potential in reducing both the incidence and mortality of lung cancer [56]. Research on salidroside has revealed its inhibitory effects on lipopolysaccharide-induced proliferation and metastasis of non-small cell lung cancer. Salidroside achieves this by suppressing the activation of the NLRP3 inflammasome. These findings indicate that NLRP3 inflammasome-mediated inflammation plays a crucial role in the proliferation and metastasis of lung cancer. Furthermore, aging is implicated in the upregulation of various inflammatory factors, including the NLRP3 inflammasome [57].

Moreover, research has demonstrated a correlation between the occurrence and development of lung cancer and the concentration of IL-1β. Low concentrations of IL-1β have been shown to stimulate the production of anti-cancer factors and activate the body's inherent immune system against cancer. Conversely, elevated concentrations of IL-1β directly contribute to the proliferation, differentiation, and metastasis of cancer cells to other tissue cells [58]. Future investigations could delve into the intricate relationship between IL-1β levels and cancer progression, providing a theoretical basis for the development of drugs modulating NLRP3 inflammasome.

#### Breast cancer

Breast cancer stands out as one of the most malignant tumors impacting women. Its pathogenesis intertwines with factors such as inflammation, age, estrogen levels, and breast tissue density. In the context of inflammation, the normal inflammatory processes become dysregulated during cellular aging, resulting in cellular damage and the prolonged release of pro-inflammatory factors. The accumulation of damaged cells within the tissue can precipitate cancerous transformations within the tissue landscape [59].

In an animal experiment in a mouse model of breast cancer, Wallenstein et al. demonstrated that the deletion of a human tumor suppressor gene (p53) in cancer cells induces TAMs to produce IL-1β, thereby promoting systemic inflammation and drive breast cancer metastasis [60]. Jin et al. reported that the NLRP3 inflammasome can be induced and activated by P2Y purinergic receptor 2 (P2Y2R) in metastatic breast cancer cells, enhancing tumor invasion and tumor growth, and contributing to tumor progression [61]. Moreover, anatomical experiments analyzing 53 breast tumor patients revealed a significant upregulation of gene expression in the NLRP3 inflammasome pathway in human breast tumor stroma compared to normal tissue stroma, suggesting an non-negligible role for this pathway in tumor progression [62]. However, the mechanism by which NLRP3 inflammasome regulate tumor progression is currently unknown.

The abnormal activation of the NLRP3 inflammasome, which may promote cancer occurrence, has spurred extensive clinical research on the development of NLRP3 inflammasome inhibitors. Among these, inhibitors targeting the NLRP3 inflammasome by blocking the IL-1 signaling pathway prove effective in breast cancer treatment. Inhibitors like anakinra and rilonacept have demonstrated promise in clinical trials [63]. Results from these trials indicate that these inhibitors significantly reduce tumor volume, inhibit growth, and alleviate symptoms and discomfort in patients. This introduces a new treatment choice for breast cancer patients, potentially enhancing their survival and quality of life.

In conclusion, inflammation emerges as a dominant force in promoting breast cancer occurrence, and NLRP3 inhibitory drugs, studied extensively to impede inflammasome activation, provide a new direction and hope for breast cancer treatment. However, further studies are necessary to confirm the safety and efficacy of these drugs in breast cancer treatment.

#### Colorectal cancer

Chronic inflammation stands out as a pivotal factor in the development of colitis-associated colorectal cancer. The neurotransmitter 5-hydroxytryptamine (5-HT) emerges as a promoter of NLRP3 inflammasome generation, initiating chronic intestinal inflammation. This sets in motion a positive feedback loop, as the NLRP3 inflammasome, in turn, mediates IL-1β maturation and catalyzes 5-HT biosynthesis by inducing tryptophan hydroxylase 1 synthesis, thereby elevating 5-HT levels in intestinal epithelial cells [64].

In the context of colorectal cancer, Wang et al. observed heightened NLRP3 expression in mesenchymal-like colon cancer cells (SW620). They demonstrated that TNF-α and transforming growth factor-β1 (TGF-β1) could upregulate NLRP3 expression in colon cancer epithelial cells, contributing to the epithelial-mesenchymal transition process—a critical facet of cancer cell metastasis. When NLRP3 was downregulated in colorectal cancer cells, a reduction in cell migration and invasion was noted [65]. However, using a colorectal cancer metastasis model to the liver, Maryse Dagenais et al. discovered that cancer-induced NLRP3 activation stimulates NK cell responses via IL-18, potentially enhancing tumor clearance [66].

The intricate relationship between NLRP3 inflammasome activation and cancer progression poses numerous areas for investigation. Key questions include whether NLRP3 inflammasome activation promotes cancer progression, its specific impact on various cancers, and the mechanisms through which inhibiting NLRP3 inflammasome activation mediates cancer progression—all warranting further exploration.

### Arthritis

**Fig. 5:**
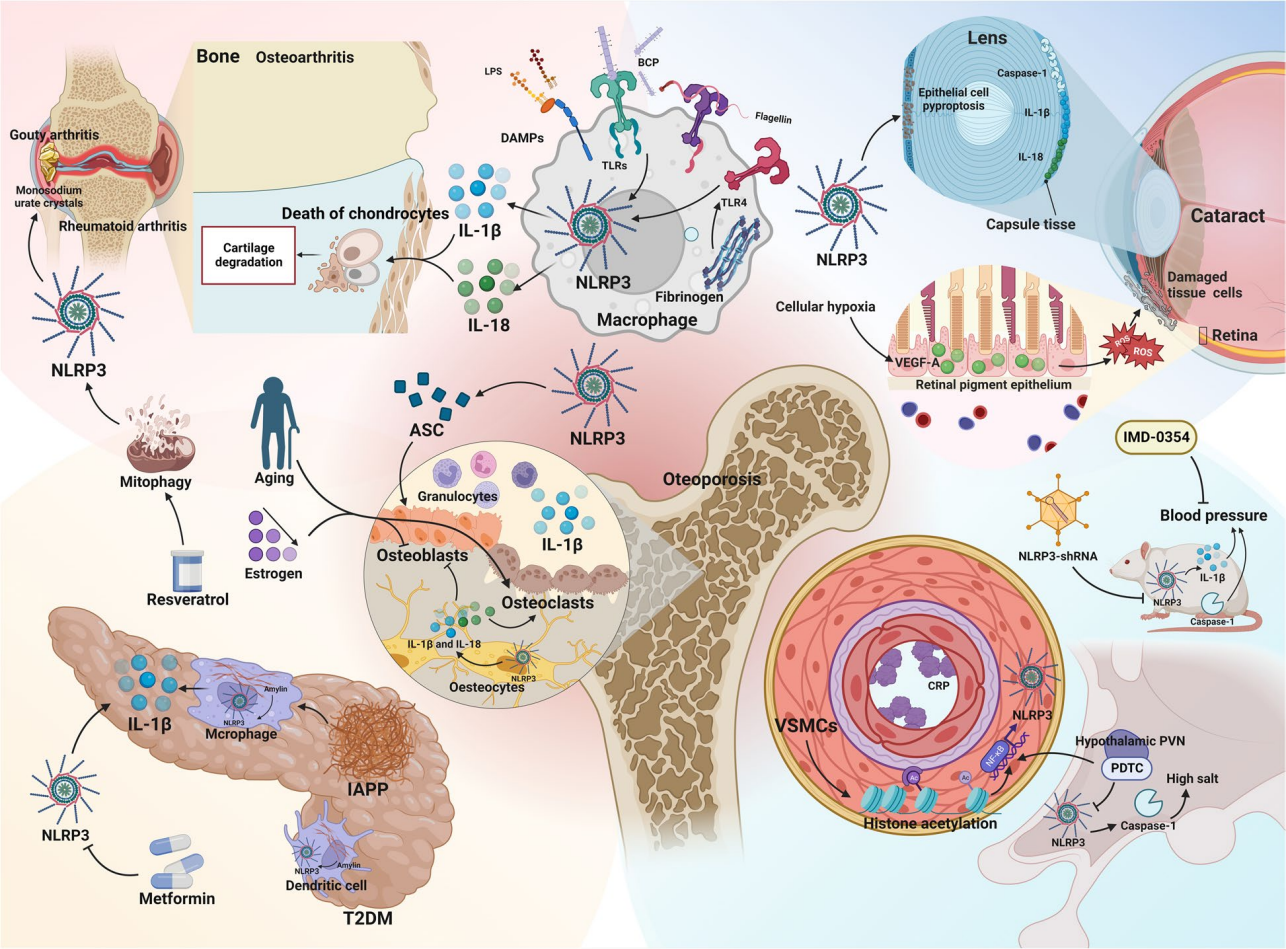
NLRP3 inflammasome in the progression of certain other aging-related diseases. BCP, basic calcium phosphate; caspase-1, cysteine-aspartic protease 1; CRP, C-reactive protein; DAMPs, damage-associated molecular patterns; IAPP, islet amyloid polypeptide; IL, interleukin; LPS, lipopolysaccharides; pro-IL-1β, pro-interleukin-1β; PDTC, pyrrolidine dithiocarbamate; PVN, paraventricular nucleus; SHR, spontaneously hypertensive rats; T2DM, type 2 diabetes mellitus; TLR4, toll-like receptor 4; TLRs, toll-like receptors; VEGF-A, vascular endothelial growth factor A; VSMCs, vascular smooth muscle cells

There is substantial evidence linking chronic or excessive inflammation to the development of arthritis [67]. Osteoarthritis, a prevalent degenerative joint disease affecting nearly any joint, exhibits elevated levels of DAMPs, notably basic calcium phosphate, in affected joints compared to normal joints. These DAMPs activate NLRP3, prompting the release of IL-1β and IL-18 from activated macrophages into the synovial fluid. This amplifies the inflammatory response cascade, culminating in chondrocyte tissue death and cartilage degeneration [68] (Fig. 5). In rheumatoid arthritis, an autoimmune disorder, TLR activation by DAMPs induces NLRP3 inflammasome expression and IL-1β release from macrophages [69]. Furthermore, stimulation of macrophages with citrulline-containing fibrinogen could indirectly stimulate NLRP3-induced IL-1β release through activation of TLR4 [70] (Fig. 5). Gouty arthritis is also an inflammatory disease in which severe pain is caused by the deposition of many monosodium urate crystals in the joints and surrounding tissues. In a drug experiment on resveratrol, the researchers revealed that resveratrol inhibited NLRP3 inflammasome activation by promoting mitophagy in a rat arthritis model, ultimately ameliorating arthritis symptoms [71] (Fig. 5).

While extensive research confirms NLRP3 inflammasome involvement in the pathophysiology of arthritis and identifies potential inflammatory mechanisms, some interventions targeting these pathways have not transitioned successfully into clinical treatments. Further exploration is essential to grasp the potential simultaneous activation of multiple pathways driving joint inflammation, paving the way for more effective therapeutic strategies.

### Cataract

Aging constitutes a significant risk factor for the development of cataracts. Over time, the lens accumulates various harmful irritants, triggering chronic inflammation in the eye. The NLRP3 inflammasome is an indispensable factor in mediating inflammation, and its massive secretion can result in cellular self-damage, contributing to the gradual loss of lens transparency [72]. Clinical experiments conducted by Deng et al. demonstrated that cataracted eyes of 70 patients released inflammatory factors, including the NLRP3 inflammasome, through the induction of epithelial cell pyroptosis [73]. Similarly, Sun et al. observed significantly increased concentrations of key markers such as active caspase-1, IL-1β, and IL-18 in the capsule tissue of cataract patients [74].

Additional studies have highlighted the occurrence of cellular hypoxia and oxidative damage in various tissues with age. These processes induce the expression of vascular endothelial growth factor A (VEGF-A) in the retinal pigment epithelium. VEGF-A, in turn, induces ROS production, leading to the release of inflammatory mediators from the NLRP3 inflammasome, exacerbating cellular and tissue damage. This interaction establishes a detrimental cycle that intensifies inflammatory responses and tissue damage. Ultimately, the increasing number of damaged cells contributes to the gradual loss of lens transparency, leading to the development of cataracts [75]. In summary, cataract development is intricately linked to chronic inflammation-induced cell pyroptosis, resulting in reduced lens transparency (Fig. 5).

### Osteoporosis (OP)

OP is a systemic bone metabolic disease and a prevalent cause of illness and mortality among the elderly [76]. For a long time, it has been widely believed that OP is an inevitable result of aging. Aging and reduced estrogen levels drive low-grade inflammation in the body, and the production of pro-inflammatory cytokines affects the growth of osteoblasts and osteoclasts, ultimately stimulating the development of OP [77]. Notably, the latest research results show that OP is preventable and treatable, which depends on the discovery of novel mechanisms contributing to its development [78].

NLRP3 plays a dual role in bone metabolism (Fig. 5). On one hand, as the adaptor protein ASC is crucial in osteoblast development, the NLRP3 inflammasome and its associated proteins actively regulate bone growth and development [79]. On the other hand, overactivation of the NLRP3 inflammasome causes aging-related osteopenia. In OP mouse models, notable symptoms of granulocyte infiltration and significantly elevated IL-1β levels were observed [80]. Within osteocytes, the NLRP3 inflammasome inhibits osteoblast activation and accelerates osteoclast differentiation through the activation of IL-1β and IL-18, leading to OP.

Overall, the NLRP3 inflammasome exerts a more significant impact on osteoclasts than osteogenesis in osteocytes. Consequently, reducing the expression of NLRP3 in osteocytes emerges as a crucial strategy to decelerate the progression of OP [76]. Given the dual role of the NLRP3 inflammasome in OP pathogenesis, research and trials involving targeted therapy inhibitors warrant careful consideration.

### Diabetes

Sterile inflammation emerges as a pivotal factor in the aging process, and accumulating evidence suggests its involvement in insulin resistance among older adults [81]. Diabetes, characterized by elevated blood sugar levels resulting from defects in insulin secretion or action, stands as one of the most prevalent metabolic diseases globally, accompanied by severe complications.

A key pathophysiological process in the development of T2DM is insulin resistance, closely entwined with inflammatory factors such as IL-1β, where the NLRP3 inflammasome assumes a central role [82]. Islet amyloid polypeptide (IAPP), or amyloid, does not form active amyloid aggregates in mice. Leveraging this characteristic, a transgenic mouse model overexpressing human IAPP was generated, revealing the induction of IL-1β production by macrophages in pancreatic islets in vivo. Another hallmark of T2DM is the accumulation of substantial amylin in the pancreas, which, upon uptake by macrophages and dendritic cells, activates the downstream NLRP3 inflammasome [83] (Fig. 5).

Moreover, metformin, the primary drug for T2DM treatment, exhibits its efficacy by reducing IL-1β levels through NLRP3 inflammasome inhibition, thereby alleviating symptoms in patients. This underscores the pivotal role of the NLRP3 inflammasome in driving the onset and progression of T2DM [84].

### Hypertension

Hypertension is a prevalent condition, particularly affecting the middle-aged and elderly population, where aging stands out as a significant contributing factor. Chronic inflammation is recognized as a common mechanism associated with aging, and C-reactive protein, an inflammatory biomarker, consistently exhibits elevated concentrations with age in the absence of overt infection [85]. Studies indicate a higher plasma concentration of C-reactive protein in hypertensive patients compared to their normal counterparts, suggesting an association between hypertension pathogenesis and inflammation [86, 87].

Using spontaneously hypertensive rats (SHR) and primary active vascular smooth muscle cells (VSMCs) from hypertensive vascular smooth muscle cells, Sun et al. investigated that in SHR-derived VSMCs, increased histone acetylation promotes nuclear factor kappa-B (NF-κB) activation, subsequently activating the NLRP3 inflammasome. Intravenous injection of NLRP3-shRNA adenovirus can down-regulate NLRP3 protein, resulting in decreased expression of caspase-1 and IL-1β protein in SHR, leading to reduced blood pressure [88]. Furthermore, Zhu et al. observed persistent elevation of NF-κB activation, the initiator of the NLRP3 inflammasome, in hypertensive patients [89]. Targeted therapies for hypertension, such as NF-κB inhibitors like IMD-0354, have proven effective in preventing the increase in right ventricular pressure [90]. Another example is pyrrolidine dithiocarbamate, a compound that inhibits NF-κB and can be infused into the hypothalamic paraventricular nucleus to block high salt by inhibiting the NLRP3 inflammasome and caspase-1, thereby inducing hypertension development [91]. This supplies therapeutic directions for preventing the development of hypertension at the level of the hypothalamus. Notably, NLRP3 inhibitors like MCC950 have demonstrated a reduction in blood pressure in animals with established hypertension [92].

In conclusion, while current studies establish an association between NLRP3 inflammatory vesicles and hypertensive disorders (Fig. 5), the specific mechanisms by which these vesicles regulate hypertension remain elusive.

### Neurodegenerative diseases

**Fig. 6:**
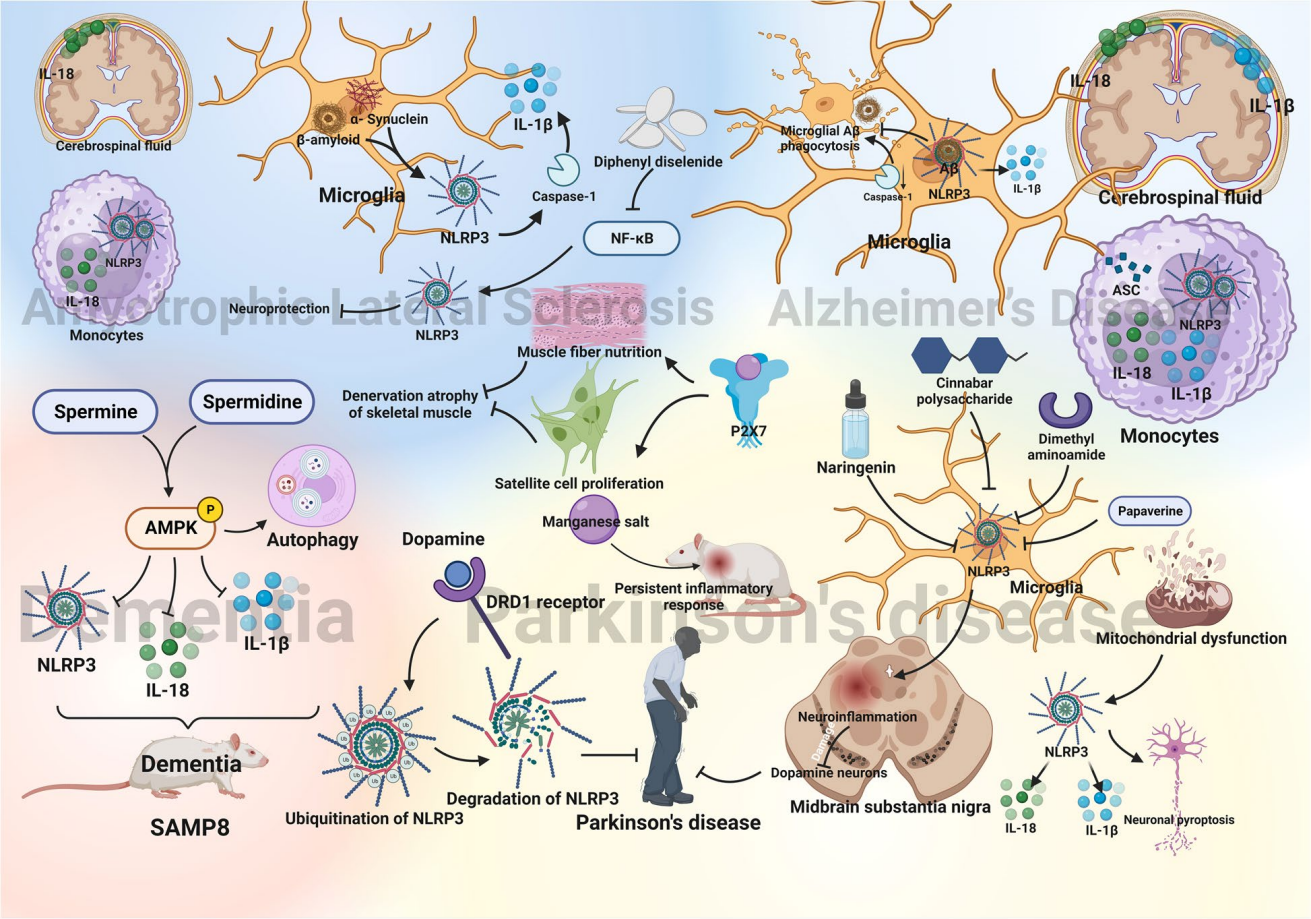
NLRP3 inflammasome in the progression of common neurodegenerative diseases. AMPK, AMP-activated protein kinase; ASC, apoptosis-associated speck-like protein; caspase-1, cysteine-aspartic protease 1; DRD1, dopamine D1 receptor; IL-18, interleukin-18; IL-1β, interleukin-1β; P2X7, recombinant purinergic receptor P2X, ligand gated ion channel 7; SAMP8, senescence accelerated mouse-8

Neurodegenerative diseases, encompassing dementia, Alzheimer's disease (AD), Parkinson's disease (PD), and amyotrophic lateral sclerosis (ALS), are primarily associated with aging. Local inflammation emerges as a common hallmark of brain aging, mirroring patterns observed in other organs and tissues [93]. Inflammasomes, particularly the NLRP3 inflammasome, exhibit activation not only in response to tissue damage and infection but also in correlation with the aging process [94]. This activation contributes to the progression of neurodegenerative diseases, the mechanisms are presented in Fig. 6.

#### Dementia

Brain aging poses a significant risk factor for dementia, with aging-induced low-grade brain inflammation playing a role in the onset of dementia. In an aging model, utilizing the senescence accelerated mouse-8 (SAMP8), Xu et al. demonstrated that spermidine and spermine exhibit potential in ameliorating aging and aging-induced dementia. These compounds were found to mediate the phosphorylation of AMPK, decrease levels of NLRP3, IL-1β, and IL-18 in vivo, induce autophagy, thereby protecting memory function and reducing brain inflammation [95]. Moreover, a study involving 79 HIV-positive patients revealed an association between increased NLRP3 inflammasome gene expression and the severity of cognitive impairment [96]. This observation underscores the impact of the NLRP3 inflammasome on the nervous system, contributing to the development of dementia.

#### Alzheimer’s disease

Alzheimer's disease, one of the most common age-associated neurodegenerative diseases in the world, is characterized by the accumulation of amyloid beta peptide (Aβ), initiating microglial activation and subsequent neuroinflammation in the brain. Microglia, the primary innate immune cells in the brain, can promote the release of inflammatory molecules for cell repair and clearing unwanted debris. The pathogenesis of Alzheimer's disease is closely linked to microglial involvement, as Aβ deposits induce the formation of NLRP3 inflammasome in microglia, leading to the maturation of interleukin-1β and triggering inflammatory responses [97].

Experiments by Michael T. Heneka et al. demonstrated that NLRP3 or caspase-1 deficiency enhanced microglial Aβ phagocytosis, while activation of NLRP3 inhibited microglial Aβ clearance in Alzheimer's disease [97]. In the cerebrospinal fluid of Alzheimer's disease patients, abnormally elevated levels of IL-1β and IL-18 were detected [98]. Furthermore, monocytes isolated and cultured from Alzheimer's disease patients exhibited increased gene expression of NLRP3, along with the cytokines IL-1β and IL-18, indicating heightened peripheral NLRP3-mediated immune responses in Alzheimer's disease progression [99].

In summary, the activation of microglial NLRP3 inflammasome by amyloid contributes to the release of pro-inflammatory cytokines, fostering the development of Alzheimer's disease pathology.

#### Parkinson’s disease

Age constitutes a significant risk factor for neurodegenerative diseases, including Parkinson's disease. As the body's organ systems undergo aging, the aging immune system contributes to inflammation. In contrast to Alzheimer's disease, Parkinson's disease not only involves the secretion of inflammatory factors by microglia but also incorporates monocytes' release of related inflammatory factors [100].

In animal experiments, Fan et al. demonstrated that the NLRP3 inflammasome directly promotes Parkinson's disease onset. They induced chronic manganese poisoning by injecting a batch of rats with a manganese salt solution every ten days for 150 days, triggering a persistent inflammatory response. As a result, these rats developed various degrees of Parkinson's disease-like syndrome [101]. Conversely, Chen et al. utilized naringenin injections in rats to prevent neuroinflammation by inhibiting the activation of the NLRP3 inflammasome in microglia. This inhibition successfully protected dopamine neurons in the midbrain substantia nigra, suppressing NLRP3 inflammasome-mediated Parkinson's disease development [102]. NLRP3 inflammasome inhibitors like cinnabar polysaccharide, papaverine, and dimethyl aminoamide have demonstrated similar effects [103–105]. Mitochondrial dysfunction, a key regulator, activates NLRP3, leading to the release of IL-1β and IL-18, inducing neuronal pyroptosis in the substantia nigra [39]. In addition, Sarkar et al. found in 2017 that the impairment of mitochondrial function in microglia enhances NLRP3 inflammasome activity [106]. Interestingly, Yan et al. discovered that dopamine promotes its degradation through dopamine D1 receptor-mediated ubiquitination of NLRP3 [107]. Consequently, the absence of dopamine may enhance NLRP3 activation, triggering Parkinson's disease, and NLRP3 ubiquitination emerges as a promising target.

#### Amyotrophic Lateral Sclerosis (ALS)

The neuromuscular junction, vital for muscle contraction control, experiences a decline in function with age, potentially contributing to motor neuron diseases, including ALS [108].

Like other neurodegenerative diseases, ALS involves an inflammatory-mediated mechanism triggered by microglial NLRP3 inflammasome activation in response to pathogenic protein aggregates such as β-amyloid and α-synuclein. This activation leads to caspase-1 activation, IL-1β secretion, and promotes ALS development [109]. In ALS patients, elevated inflammatory markers and IL-18 levels in serum and cerebrospinal fluid, along with increased NLRP3 inflammasome and IL-18 expression in monocytes, underscore the involvement of the NLRP3 pathway [110, 111]. Notably, Zhang et al. demonstrated the neuroprotective potential of diphenyl diselenide by inhibiting the NF-κB pathway and NLRP3 inflammasome activation, suggesting it as a promising treatment for ALS [112]. In an animal experiment, the researchers used SOD1G93A mice to mimic some of the pathological features of ALS to study the possible effects of activating the P2X7 receptor in skeletal muscle. The results suggest that activation of the P2X7 receptor improves muscle fiber nutrition and promotes satellite cell proliferation, thereby preventing denervation atrophy of skeletal muscle, which inhibits ALS progression to some extent [113]. This example highlights the potential to inhibit ALS development by safeguarding the neuromuscular junction from factors such as the NLRP3 inflammasome.

The evidence underscores the pivotal role of the NLRP3 inflammasome in AD, PD, and ALS, warranting further exploration as a therapeutic target.

## Conclusion

During aging, the activation and hyperactivation of the NLRP3 inflammasome during aging have been closely associated with inflammatory responses and cellular damage. Studies have demonstrated that NLRP3 inflammasome activation triggers intracellular inflammation, contributing to the aging process. Furthermore, its activation is intricately linked to the onset and progression of diverse age-related diseases, including cardiovascular diseases, cancer, arthritis, cataracts, OP, diabetes, hypertension, and neurodegenerative diseases.

Going forward, it is important to further study the role and regulation mechanism of NLRP3 inflammasome in aging and age-related diseases. A deep understanding of the function and regulatory pathways of the NLRP3 inflammasome can supply an important theoretical basis for the development of new therapeutic strategies and drugs. In addition, researchers can further explore the interaction of NLRP3 inflammasome with other inflammasomes, as well as the cross-regulation mechanism with other related signaling pathways. This study will help in-depth understanding of the mechanisms of aging and age-related diseases and supply innovative ideas and methods for the prevention and treatment of these diseases.

## Data Availability

Not applicable.
